# A cross-sectional study of functional movement quality in school-aged children

**DOI:** 10.1186/s12887-022-03410-2

**Published:** 2022-07-07

**Authors:** Sajad Bagherian, Khodayar Ghasempoor

**Affiliations:** 1 Department of Sport Sciences, Shahrekord University, Shahrekord, Iran; 2 Department of Physical Education and Sport Sciences, Technical and Vocational University, Tehran, Iran

**Keywords:** Fusionetics, Functional movement screen, School-aged children, Movement quality

## Abstract

**Background:**

During the growth period, before and after maturity, considerable biological changes occur. It seems that these changes are related to neuromuscular patterns and have significant differences in the functional movements performed of young boys and girls during the maturation process. The current study aimed to look at the movement quality scores of school-aged girls and boys.

**Methods:**

This Cross-Sectional Study assessed the movement quality of 700 school-aged boys and girls aged 8 to 17, divided into 10 groups of 35 girls and 10 groups of 35 boys. Movement quality was evaluated by the Fusionetics scoring system, which includes 7 tasks: two-leg squat, two-leg squat with heel raise, one-leg squat, push-up, shoulder, trunk, and cervical movements that require a person to complete different movement patterns. The data was analyzed using the Wilcoxon signed-rank and McNemar tests (*p* <0.05).

**Results:**

This is the first study to our knowledge to examine the movement quality scores in a large school age child with Fusionetics. The overall results showed that the most errors were recorded in all age groups during the double leg squat, double leg squat with heel lift, single leg squat, and push-up and school-age children showed less errors during the shoulder movements, trunk/lumbar spine movements and cervical spine movements. Furthermore, younger girls and boys made more errors than older girls and boys. In relation to gender, this study found that girls scored better on the total Fusionetics score than boys.

**Conclusions:**

The Fusionetics scoring system explains how well school-aged children perform fundamental movements. Under the guidance of coaches and physical educators, students' movement compensation should be assessed and relevant training interventions implemented. Taking steps to address movement compensation could help to avoid injuries and improve school-age children performance.

**Supplementary Information:**

The online version contains supplementary material available at 10.1186/s12887-022-03410-2.

## Introduction

Several performance-based and movement-competency-based tests for identifying neuromuscular capacity deficits associated with increased injury risk have recently been identified. The Functional Movement Screen (FMS) is a movement-competency-based measure that is commonly used in clinical practice [1]. The value of FMS in child development is supported by conclusive evidence [2]. FMS proficiency has been shown in recent decades to be critical for children's physical (i.e. cardiorespiratory fitness, healthy weight) and psychosocial (i.e. physical self-concept) wellbeing [2]. FMS proficiency has also been shown to have a positive relationship with children's involvement in physical activity [3]. There is also evidence for a reversal mechanism, in which childhood obesity leads to motor proficiency declines [4]. The FMS was also used in other studies to look into the connection between BMI, physical activity, and functional movement in children. According to the authors, poor functional movement was related to a higher BMI and lower levels of physical activity [5].

Different terminology has been used inconsistently throughout literature in the study of movement characteristics in children, such as "movement competence," "motor competence," "fundamental movement skills," "motor proficiency," and "motor skill." All of these terms refer to the study of “fundamental motor skills,” which are described as the global movement patterns (such as locomotion, object control, or stability tasks) that are required for optimal motor growth [6]. The term "movement efficiency" is used in this research to refer to the study of fundamental motor skills as described above. The Fusionetics method, a new evaluation of functional movement efficiency, was recently presented in the literature [7, 8]. The Fusionetics, like the FMS, has seven sub-tests (two-leg squat, two-leg squat with heel raise, one-leg squat, push-up, shoulder, trunk, and cervical movement) that require a person to complete different movement patterns [9]. The Fusionetics tasks, on the other hand, are graded on the existence of unique movement compensations (errors) that are seen frequently during each subtest [9]. Fusionetics uses computer-based proprietary algorithms to generate a 0–100 (worst–best) score for each individual sub-test based on movement compensations detected throughout the whole evaluation [9]. There has been no research into the movement patterns of school-aged children using the Fusionetics method to our knowledge. The goal of this study was to use the Fusionetics method to look at different movement patterns in school-aged boys and girls. We hypothesized that school-aged children would perform differently in terms of movement.

## Methods

### Design

A cross-sectional study was conducted following the Strengthening the Reporting of Observational Studies in Epidemiology (STROBE) recommendations [10]. Written informed consent was obtained from the participants’ parents or guardians. Ethical approval was obtained from the research ethics committee of the University of Tehran before performing the study (IR.UT.SPORT.REC.1398.012).

### Subjects

The sample size was determined using the Cochran formula (a= 0.05, power=80%, 95% CI). We required 380 subjects for both boy and girl school-age children because the population of boy and girl school-age children (primary, secondary, and high schools) in Shahrekord city, Iran was 35000. A stratified random sampling of all primary, secondary, and high schools was performed. One or two classes from each of the grades 2, 3, 4, 5, 6, 7, 8, 9, and 10 based on the population of each class were chosen at random within each selected school. Finally, a total of 700 healthy school-aged children, aged 8 to 17, were included in the study, divided into 10 groups of 35 girls and 10 groups of 35 boys (Table 1). Health doctors had cleared all of the participants to take part in the study, and none of them had been injured or had a history of injury in the previous 6 months.

**Table 1:** Characteristics of school-age children

Age Group	Age (year)		Weight		Height		BMI	
	Boys	Girls	Boys	Girls	Boys	Girls	Boys	Girls
8+	8.45± 0.50	8.54± 0.50	26.5 ± 3.9	28.1 ± 7.7	134.3 ± 5.1	132.3 ± 6.1	14.5 ± 1.9	15.9 ± 3.6
9+	9.57± 0.50	9.60± 0.49	31.3 ± 7.8	31.4 ± 8.9	138.2 ± 6.5	138.1 ± 6.1	16.3 ± 3.1	16.1 ± 3.4
10+	10.57± 0.55	10.45± 0.50	36.1 ± 10.9	38.1 ± 9.9	144.1 ± 4.8	143.1 ± 8.2	17.5 ± 4.7	17.9 ± 3.2
11+	11.48± 0.50	11.60± 0.49	37.4 ± 8.9	39.3 ± 9.7	149.3 ± 6.2	150.6 ± 7.9	16.4 ± 3.1	16.9 ± 3.1
12+	12.51± 0.50	12.28± 0.51	46.1 ± 12.4	43.2 ± 8.9	157.2 ± 9.1	154.3 ± 5.4	18.5 ± 4.2	18.5 ± 3.7
13+	13.40± 0.49	13.62± 0.54	52.2 ± 11.6	53.4 ± 14.2	162.3 ± 7.1	157.1 ± 7.8	19.7 ± 4.1	21.6 ± 4.8
14+	14.65± 0.63	14.45± 0.50	58.2 ± 12.4	54.3 ± 14.2	166.1 ± 7.9	157.5 ± 10.9	20.9 ± 3.7	21.6 ± 4.5
15+	15.65± 0.48	15.62± 0.54	60.1 ± 11.8	60.5 ± 13.4	173.2 ± 5.2	167.2 ± 4.9	19.9 ± 3.4	21.6 ± 4.3
16+	16.48± 0.50	16.40± 0.55	66.1 ± 11.9	59.2 ± 11.5	177.2 ± 6.7	166.4 ± 6.3	20.9 ± 3.6	21.5 ± 4.4
17+	17.48± 0.50	17.40± 0.49	68.3 ± 13.3	58.1 ± 10.3	177.1 ± 5.9	165.1 ± 5.2	21.5 ± 3.6	21.1 ± 3.3

### Study overview

For both boys and girls, all data was collected under normal conditions over a two-week period. The students were taken from their school in small groups to a public sports center, where they were assessed in an indoor facility.

The Fusionetics Scoring System (FSS, Fusionetics®, Milton, GA, USA) was used to evaluate the movement efficiency scores based on the company’s proprietary scoring algorithms. The proprietary scoring algorithm takes into consideration the number of errors, the type of errors, and the body region where the error occurred [7, 8]. As a test for functional movement quality, the Fusionetics had excellent intra-rater test-retest reliability [9].

The movement efficiency tasks were also performed according to Fusionetics' instructions (www.fusionetics.com). In brief, all participants completed the movement efficiency assessments while dressed in athletic clothing and without shoes. Each participant also completed the following sub-tests in the following order: two-leg squat, two-leg squat with heel raise, one-leg squat, push-up, shoulder, trunk, and cervical movement. Appendix A contains more detailed explanations of the tasks, movement instructions, and movement compensations (errors) for each task. The participants were also given five trials of each sub-test, with the most proficient trial (i.e., the one with the least compensation) being used for scoring [9, 11].

Each sub-test was scored in real time in a binomial (Yes/No) manner based on a standard set of movement compensations seen during each sub-test (Appendix B). In total, 60 compensations were scored across all sub-tests of the movement efficiency tests. After scoring each sub-test, these binomial data were entered into the Fusionetics Scoring System. This online platform utilizes a proprietary algorithm to calculate a movement efficiency test score for the overall assessment (i.e., the Overall movement efficiency test score), as well as a movement efficiency test score for each individual sub-test. These movement efficiency test scores are considered interval-level data and range from 0 to 100 (viz. worst to best) [9, 11]. The research team performed both online training and repeated scoring of 10 pilot participants until an appropriate degree of reliability was reached before beginning this investigation.

### Statistical Analyses

The data were analyzed with the Statistical Package for Social Sciences (SPSS) version 26 (IBM Inc., Chicago, IL, USA) and *p*-value <0.05 was considered as statistical significance for all assessments. Fusionetics scores for each task, as well as total Fusionetics scores for all seven tasks, were compared between boys and girls using the Wilcoxon signed-rank test. Since the movement efficiency scores (errors) were qualitative data, the McNemar test was used to compare movement errors between boys and girls.

## Results

The percentage of participants making a given error (e.g., knee valgus) during each task was recorded and reported in Table 2. For example, in the "Foot Turns Out", 8-year-old children demonstrated a significant difference between right and left foot for each gender (*P*<0.05), and there was a significant difference between the left feet of boys and girls (*P*<0.05), but not the right foot (*P*>0.05).

**Table 2:**
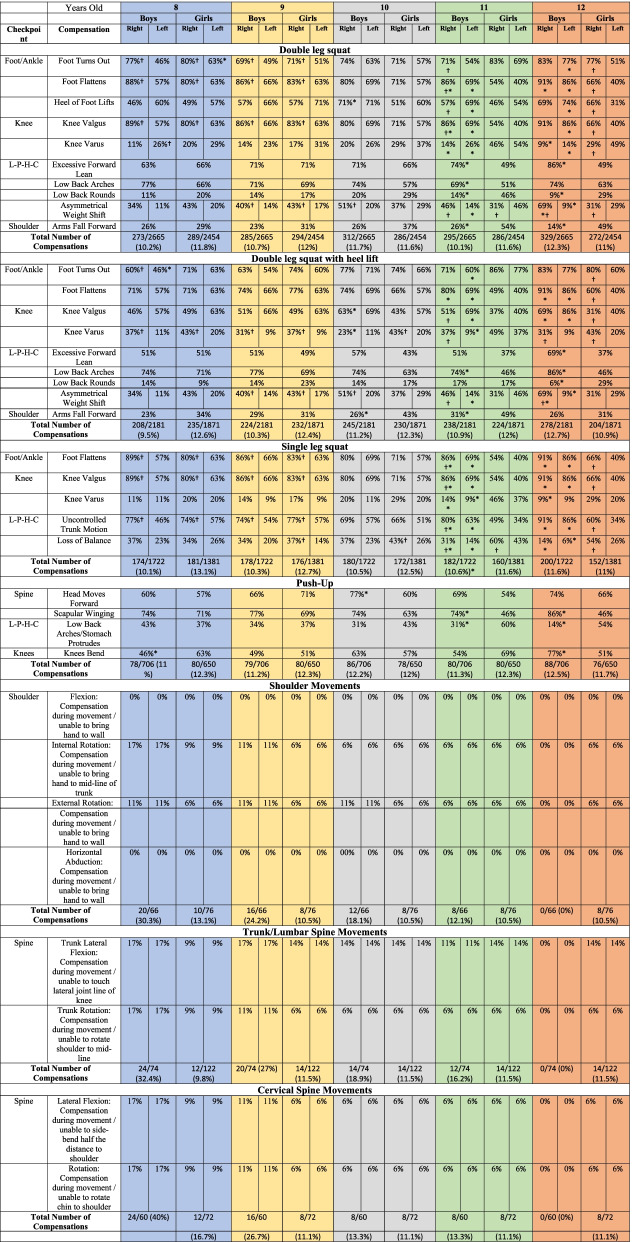

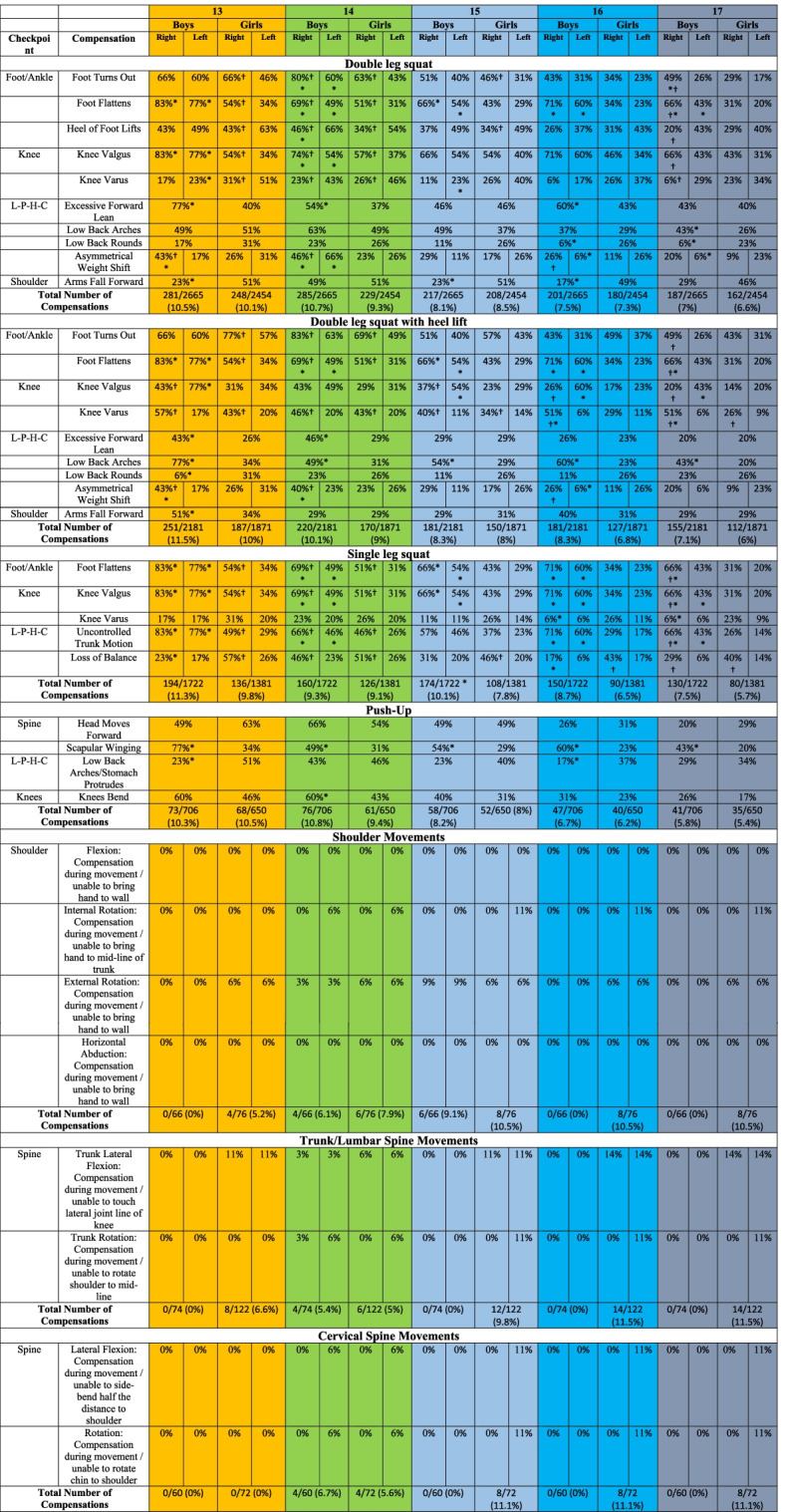
Boys’ and girls' specific movement compensations (errors) by age group

*L-P-H-C* Lumbo-pelvic-hip complex

*Notes* Data are presented as the percentage of participants that committed a specific compensation. The total number of compensations for each task by age group are also presented

Items that can be scored for a right and left limb are presented under the right and left columns for each age group. Items scored for the entire body (e.g. low back arches) are presented as a singular score under each age group

Significant differences between boys and girls for each error are indicated by an asterisk (*), and significant differences between right and left side errors for each gender are indicated by a dagger (†) (*p*<0.05)

Tables 2 also indicate the total amount of compensation for both boys and girls for each task. For example, in the "Double leg squat," boys and girls made 273 and 289 errors, respectively, in terms of total compensation for 8-year-old children. The most errors were recorded in all age groups during the double leg squat, double leg squat with heel lift, single leg squat, and push-up and school-age children showed fewer errors during the shoulder movements, trunk/lumbar spine movements and cervical spine movements Table 2.

**Fig. 1:**
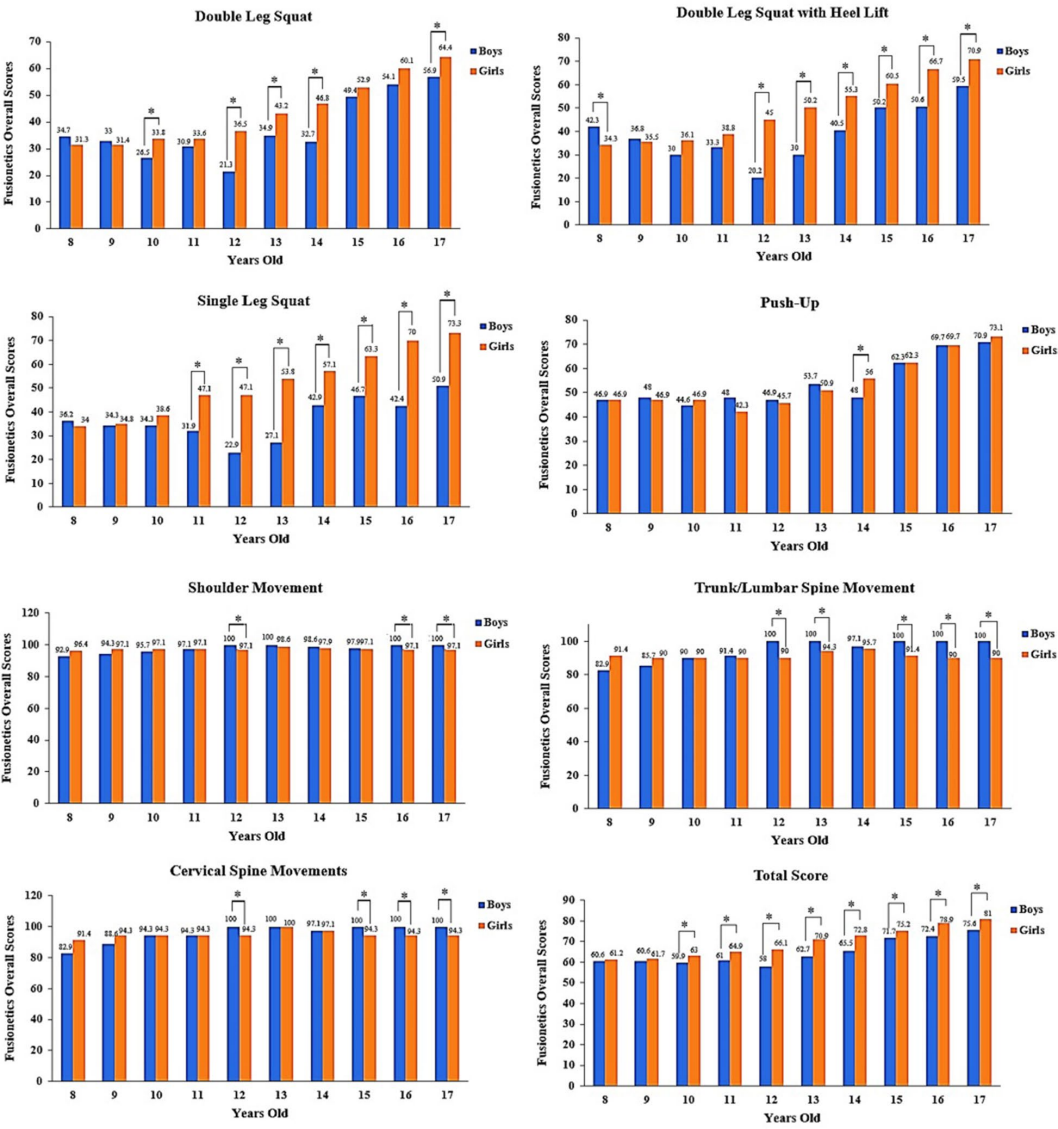
Fusionetics scores for each task, as well as the total Fusionetics scores for all tasks

Fusionetics overall scores for each task, as well as overall Fusionetics scores for all seven tasks, were reported in Table 3. Overall, younger girls and boys made more errors than older girls and boys Table 3. Figure 1 shows the Fusionetics scores for each task, as well as the total Fusionetics scores for all tasks for each age group and gender combined from 0 to 100 (viz. worst to best). In relation to gender, the results of this study revealed that girls had a higher (better) total Fusionetics score in all age groups and all tasks, particularly in squats (double leg squat, double leg squat with heel lift, single leg squat), which was significant for the age range of 12 to 17 years old (Fig. 1). Boys showed a better overall Fusionetics score in shoulder (12, 16 and 17 years old), trunk/lumbar spine (12, 13, 15-17 years old), and cervical spine (12, 15-17 years old) movements (Fig. 1).

**Table 3:** Fusionetics overall scores for each task, as well as overall Fusionetics scores for all seven tasks, were calculated for each age group

	**Double Leg Squat**		**Double Leg Squat with Heel Lift**		**Single Leg Squat**		**Push-Up**	
**Age Group**	**Boys**	**Girls**	**Boys**	**Girls**	**Boys**	**Girls**	**Boys**	**Girls**
**8**	34.73	31.36	42.32*	34.32	36.19	34.05	46.86	46.86
**9**	33.05	31.46	36.82	35.46	34.29	34.76	48.00	46.86
**10**	26.51*	33.81	30.06	36.16	34.29	38.57	44.57	46.86
**11**	30.86	33.62	33.27	38.82	31.91*	47.14	48.00	42.29
**12**	21.27*	36.54	20.25*	45.05	22.86*	47.14	46.86	45.71
**13**	34.89*	43.18	29.97*	50.22	27.14*	53.81	53.71	50.86
**14**	32.67*	46.83	40.51*	55.30	42.86*	57.14	48.00*	56.00
**15**	49.40	52.95	50.22*	60.48	46.67*	63.33	62.29	62.29
**16**	54.12	60.16	50.60*	66.70	42.38*	70.00	69.71	69.71
**17**	56.86*	64.45	59.49*	70.89	50.95*	73.33	70.86	73.14
	**Shoulder Movement**		**Trunk/Lumbar Spine Movement**		**Cervical Spine Movements**		**Total Score**	
**Age Group**	**Boys**	**Girls**	**Boys**	**Girls**	**Boys**	**Girls**	**Boys**	**Girls**
**8**	92.86	96.43	82.86	91.43	82.86	91.43	60.64	61.22
**9**	94.29	97.14	85.71	90.00	88.57	94.29	60.59	61.69
**10**	95.71	97.14	90.00	90.00	94.29	94.29	59.86*	63.05
**11**	97.14	97.14	91.43	90.00	94.29	94.29	60.99*	64.91
**12**	100.00*	97.14	100.00*	90.00	100.00*	94.29	58.00*	66.17
**13**	100.00	98.57	100.00*	94.29	100.00	100.00	62.70*	70.90
**14**	98.57	97.86	97.14	95.71	97.14	97.14	65.51*	72.81
**15**	97.86	97.14	100.00*	91.43	100.00*	94.29	71.68*	75.25
**16**	100.00	97.14	100.00*	90.00	100.00*	94.29	72.39*	78.88
**17**	100.00*	97.14	100.00*	90.00	100.00*	94.29	75.62*	80.99

*Note*: Asterisk (*) indicates significant differences between boys and girls (*p*<0.05).

Scores range from 0 to 100. (viz. worst to best)

## Discussion

The aim of this study was to conduct an overview of functional movement quality in school-aged children. The overall results showed that most errors recorded in all age groups occurred during the double leg squat, double leg squat with heel lift, single leg squat, and push-up. This suggests that more errors are made in the tasks that require more effort (squats, push-ups). Further, the tasks that required greater effort revealed more muscle imbalances. To figure out about muscle imbalance in individuals, there are different ways, such as static and dynamic assessments [12]. As a result of muscle imbalance such as poor neuromuscular control and poor dynamic stability of the trunk and lower extremities, dynamic malalignments (e.g., knee valgus) can occur during functional movements (e.g., squatting) [9, 11]. Fusionetics tasks are dynamic assessments which assume there are muscle imbalances based on the scores during functional tasks. In dynamic conditions, static malalignments (altered length-tension relationships caused by poor static posture, joint dysfunction, and myofascial adhesions) have been reported to cause abnormal muscle recruitment patterns (altered force-couple relationships) [12], which we refer to as movement compensation (errors) in this study. According recent epidemiological studies, 68 percent of the young population has at least one static postural alterations (e.g., thoracic hyperkyphosis, lumbar hyperlordosis) [13] that may affect functional movement quality. For example, in our study, approximately 65% of school-age students showed lumbar hyperlordosis (low back arches) during performing double leg squats, whereas it was less in more mature students. In this respect, Molina-Garcia et al. [14] discovered that children with a higher total FMS score had a more aligned sagittal plane posture of the thoracic and lumbar spines. Future research should look at how static misalignments affect dynamic situations in school-aged children.

According to the findings of this study, older boys and girls made fewer errors than younger children. For example, in cervical spine movements, 8-and 9-year-old boys make 40 and 27 percent errors, respectively, while the number of errors decreases with age, and there were no errors in boys 15 years old and older. Table 2 shows the similar outcome for different tasks. It can be interpreted that during the growth period, before and after age at peak height velocity (PHV), considerable biological changes occur [15]. It seems that these changes are related to neuromuscular patterns and have significant differences in the functional movements performed of young boys and girls during the maturation process [16]. For example, it has been reported that boys reach a growth spurt or PHV at the age of 14 and girls at the age of 12 [15]. It's possible to determine that as they grow up, their movement quality improves. On the other hand, it’s possible that individuals who are more physically active tend to learn and develop functional movements more easily, especially if the children participate in structured physical activity [17]. We did not measure the physical activity of school students in this study, but other studies, such as Cliff et al. [4], advocated the idea that with a high level of performance in FMS, an increased level of physical activity may be noticed, which is supported by longitudinal studies [17]. Similarly, physical activity appears to be linked to functional movement in children, supporting the hypothesis that functional movement impairment leads to greater sedentary time or vice versa [18]. It appears that as children grow, their participation in structured sports may increase, which can be attributed to better scores in the older age group. In this study, we looked at the functional task in school-aged children regardless of whether or not they were overweight or obese, or how fit they were, as these factors could influence optimal movement patterns. Future research should focus on the fatness and fitness of school-aged children in relation to their growth and functional movement quality.

In terms of gender, the findings of this study revealed that girls of all ages had a better total Fusionetics score, which was especially noticeable in the age group of 10 to 17 years old (Fig. 1). In this regard, Jaakkola and Washington [17] found stable FMS correlations over time for both boys and girls, but only partially existing relationships between physical activity and FMS within a grade and over time, as well as for physical activity over time. Overall, there was no apparent trend for a gender effect: while some research indicates positive results for boys, others have been unable to substantiate such a difference [16]. These findings, however, are consistent with Burton et al. [19], who found that girls scored higher on the total FMS than boys because they performed better in the deep squat, in line lunge, straight leg raise, and shoulder rotation. Despite the fact that most study designs only assess functional movement in boys or girls, further research is needed to evaluate the quality of functional movement in both boys and girls at the same time. Furthermore, the relationship between maturity and movement efficiency requires further investigation. As a result, assessing movement efficiency should be an important part of any youth physical development program. Academies should regularly assess young athletes' movement efficiency and maturity level in order to identify those who are at a higher risk of injury.

Functional movement assessments have become more common in clinical practice because they are a quick clinician-oriented tool for identifying lower extremity injury risk factors [1].. However, in school-aged participants, only the FMS has been used to quantify functional movement scores [3, 20]. While FMS system scoring has been shown to be reliable, [21] the small range of possible scores (i.e., [1–3]) may limit the sensitivity of the FMS. More recently, the Fusionetics platform was released, which consists of seven tasks with strong intra-rater test-retest reliability: two-leg squat, two-leg squat with heel raise, one-leg squat, push-up, shoulder, trunk, and cervical movement [9]. The percentage of participants committing a certain error (e.g., foot turns out) during each task was also computed using this information Table 2. In addition, the Fusionetics Scoring System assigns a total score to each task and another total score to all tasks combined based on the occurrence of an error in a body part, that it can assist us in interpreting the quality of functional movements more accurately. To use Fusionetics to identify movement compensations in children as a longitudinal study, more research is required. It's also possible that the Fusionetics' 0–100 scoring scale will be more sensitive to changes in functional movement quality as a result of a targeted corrective exercise intervention, but more study is needed to test this hypothesis. It's also worth emphasizing that the findings don't necessarily mean that children with poor functional movement at this age need medical assistance. More study is needed to confirm these findings, and randomized controlled trials should focus on exercise intervention programs aimed at improving children's functional movement quality. Under the guidance of coaches and physical educators, students' movement compensation should be assessed and relevant training interventions implemented. Taking steps to address movement compensation could help to avoid injuries and improve school-age children performance.

### Strengths and limitations

To the best of our knowledge, this is the first Cross-Sectional Study that examines movement quality scores of school-aged girls and boys using the Fusionetics Scoring System. The methodological quality was assessed with twenty-two criteria adapted from the STROBE statements. We tried to show the influence of age and gender on functional movement quality. During this investigation, the authors were unable to collect the amount of physical activity of school students as well as lack of evaluation of other fitness indicators and their relationship with movement scores. More research is needed to determine the relationship between school students' physical activity levels and Fusionetics scores, as well as to conduct longitudinal studies on Fusionetics scores across age groups as well as the long-term effect of exercise interventions on Fusionetics scores.

## Conclusions

In conclusion, the findings of this study show that younger girls and boys made more errors than older girls and boys, and that girls outperformed boys on the total Fusionetics score. Most errors were recorded in all age groups during the double leg squat, double leg squat with heel lift, single leg squat, and push-up and school-age children showed fewer errors during the shoulder movements, trunk/lumbar spine movements and cervical spine movements. The research lines must be focused on determining the causes of movement compensations (errors) during functional tasks in school-age students. Also, the knowledge about the characteristics and abilities of children who show more movement compensation will contribute to the development of adequate training treatments that would be stimulating and have a reduced risk of injuries.

## Supplementary Information

**Additional file 1.**

**Additional file 2.**

## Data Availability

The dataset analyzed for this study is available from the corresponding author on reasonable request.
