# Supplementary material for: A cross-sectional study of functional movement quality in school-aged children
Source: BMC Pediatr. 2022 Jul 7;22:399. doi: 10.1186/s12887-022-03410-2 (PMC9264668; doi:10.1186/s12887-022-03410-2)
Supplement: Supplementary file 1 — Additional file 1. [file 12887_2022_3410_MOESM1_ESM.docx]

Appendix A. Movement Efficiency Test Checklist.

| **TEST / MOVEMENT** | **STARTING POSITION / MOVEMENT INSTRUCTIONS** | **CHECKPOINT EVALUATION /**  **OBSERVATION CRITERIA FOR FAIL** |
| --- | --- | --- |
| **2-Leg Squat** | - Feet shoulder-width apart - Toes pointing straight ahead - Arms extended directly overhead - Perform 5-15 squats as if sitting into a chair - Evaluate: Front, side, and back view | - Foot Turns Out: Any lateral deviation from starting position - Foot Flattens: 5th Metatarsal is elevated and/or toes lift - Knee Moves In: Mid-patella moves medial of 1st Toe - Knee Moves Out: Mid-patella moves lateral of 5th Toe - Excessive Forward Lean: Inability to maintain a torso parallel to the tibia - Low Back Arches: Increased lumbar extension from starting position - Low Back Rounds: Increased lumbar flexion from starting position (occurring before 90° of hip flexion) - Arms Fall Forward: Inability to maintain a straight line as an extension of the torso - Heel of Foot Lifts (2-Leg Squat only): Inability to keep heels in contact with floor - Asymmetrical Weight Shift: Record the side shifting TOWARDS |
| **2-Leg Squat with Heel Lift** | - Elevate heels approximately 2” - Feet shoulder-width apart - Toes pointing straight ahead - Arms extended directly overhead - Perform 5-15 squats as if sitting into a chair - Evaluate: Front, side, and back view |  |
| **1-Leg Squat** | - Toes pointing straight ahead - Athlete balances on 1-Leg - Place hands on hips - Perform 5 squats per leg - Evaluate: Front view | - Foot Flattens: 5th Metatarsal is elevated and/or toes lift - Knee Moves In: Mid-patella is medial of 1st Toe - Knee Moves Out: Mid-patella is lateral of 5th Toe - Uncontrolled Trunk: Inability to maintain torso parallel to the tibia and/or any change from starting/neutral position - Loss of Balance: Two or more touches with the non-involved foot and/or any hopping to retain balance |
| **Push-Up** | - Assume a push-up position w/ hands outside shoulders (thumb to armpit) and even with chest - Eyes looking toward ground - Perform 5-10 push-ups - Lower chest to within 3-5 inches from ground - Evaluate: Side view | - Head Moves Forward: Increased cervical extension and/or flexion - Scapular Winging: Any asymmetry or excessive elevation of inferior pole and/or medial border of scapula - Low Back Arches/Stomach Protrudes: Inability to maintain rigid trunk-hip-leg body posture throughout movement and/or abdominal contents distend - Knees Bend: Inability to maintain a straight-leg orientation |
| **Shoulder Movements** | - Stand with heels, butt, shoulders & back of head against wall - Feet hip-width apart, arms by sides - Flexion: Raise arm straight up, touch thumb to wall overhead - Internal Rotation: Shoulders Abducted 90°, Elbows at 90°, rotate shoulder taking hand forward toward lateral mid-line of body - External Rotation: Shoulders Abducted 90,° Elbows at 90,° rotate shoulder touching back of wrist to wall. - Horizontal Abduction: Hands together in front of body, reach back of hand to wall - Evaluate: Front & Side view - Perform 1-Arm at time | - Flexion: - Inability to touch thumb to wall - Inability to keep elbow fully extended - Inability to keep head against the wall - Early/excessive shoulder elevation - Any movement in unwanted planes - Any change in lumbar positioning - Internal Rotation: - Inability to get wrist to lateral mid-line of body - Elbow flexes or extends during movement - Inability to keep head against the wall - Shoulder protraction away from wall - External Rotation: - Inability to get back of wrist to wall and/or compensates w/ wrist extension so finger tips touch wall - Elbow flexes or extends during movement - Inability to keep head against wall - Early/excessive shoulder elevation - Any change in lumbar positioning - Horizontal Abduction: - Inability to keep head against the wall - Elbow flexes during movement - Early/excessive shoulder elevation - Any change in lumbar positioning - Trunk rotates to the involved side |
| **Trunk Movements** | - Stand with heels, butt, shoulders, & back of head against wall - Feet hip-width apart, arms by sides - Lateral Flexion: Side bend and slide hand down outside of leg to lateral aspect of knee - Rotation: Athlete steps away from wall places hands across shoulders - Rotation: Rotate upper body one direction as far as possible - Evaluate: Fonts & Side view - Perform movements in each direction | - Lateral Flexion: - Inability to reach fingers to lateral joint line - Knee flexes on involved side - Inability to maintain neutral pelvis - Opposite heel lifts off floor - Any movement in unwanted planes - Rotation: - Early movement of the pelvis to get more rotation - Low back flexes/extends and/or laterally flexes - Inability to align anterior acromion to umbilicus |
| **Cervical Movements** | - Feet hip-width apart, arms by sides - Head in neutral position - Lateral Flexion: Tip head, moving ear toward shoulder - Rotation: Rotate head and look over shoulder - Evaluate: Front & Side view - Perform movements in each direction | - Lateral Flexion: - Any movement in unwanted planes - Inability to bring ear to practitioner’s fingers - Asymmetrical lateral flexion - Rotation: - Any movement in unwanted planes - Inability to get middle of chin (cleft) to align with anterior acromion |
