# Supplementary material for: A cross-sectional study of functional movement quality in school-aged children
Source: BMC Pediatr. 2022 Jul 7;22:399. doi: 10.1186/s12887-022-03410-2 (PMC9264668; doi:10.1186/s12887-022-03410-2)
Supplement: Supplementary file 2 — Additional file 2. [file 12887_2022_3410_MOESM2_ESM.pdf]

FUSIONETICS™

MOVEMENT EFFICIENCY (ME) TEST

Name: ______________________________ Date: ____ /____ /____

2-LEG SQUAT

CHECKPOINT	COMPENSATION	RIGHT	LEFT
**VIEW: FRONT**			
Foot/Ankle	Foot Turns Out		
	Foot Flattens		
Knee	Knee Moves In (Valgus)		
	Knee Moves Out (Varus)		
**VIEW: SIDE**			
L-P-H-C	Excessive Forward Lean		
	Low Back Arches		
	Low Back Rounds		
Shoulder	Arms Fall Forward		
**VIEW: BACK**			
Foot/Ankle	Heel of Foot Lifts		
L-P-H-C	Asymmetrical Weight Shift		

2-LEG SQUAT WITH HEEL LIFT

CHECKPOINT	COMPENSATION	RIGHT	LEFT
**VIEW: FRONT**			
Foot/Ankle	Foot Turns Out		
	Foot Flattens		
Knee	Knee Moves In (Valgus)		
	Knee Moves Out (Varus)		
**VIEW: SIDE**			
L-P-H-C	Excessive Forward Lean		
	Low Back Arches		
	Low Back Rounds		
Shoulder	Arms Fall Forward		
**VIEW: BACK**			
L-P-H-C	Asymmetrical Weight Shift		

1-LEG SQUAT

CHECKPOINT	COMPENSATION	RIGHT	LEFT
**VIEW: FRONT**			
Foot/Ankle	Foot Flattens		
Knee	Knee Moves In (Valgus)		
	Knee Moves Out (Varus)		
L-P-H-C	Uncontrolled Trunk: Flexion, Rotation, and/or Hip Shift		
	Loss of Balance		

PUSH-UP

CHECKPOINT	COMPENSATION	
**VIEW: SIDE**		
Spine	Head Moves Forward	
	Scapular Winging	
L-P-H-C	Low Back Arches/Stomach Protrudes	
Knees	Knees Bend	

SHOULDER MOVEMENTS

CHECKPOINT	COMPENSATION	RIGHT	LEFT
**VIEW: SIDE**			
Shoulder	Flexion: Compensation during movement / unable to bring hand to wall		
	Internal Rotation: Compensation during movement / unable to bring hand to mid-line of trunk		
	External Rotation: Compensation during movement / unable to bring hand to wall		
	Horizontal Abduction: Compensation during movement / unable to bring hand to wall		

TRUNK/LUMBAR SPINE MOVEMENTS

CHECKPOINT	COMPENSATION	RIGHT	LEFT
**VIEW: FRONT**			
Spine	Trunk Lateral Flexion: Compensation during movement / unable to touch lateral joint line of knee		
	Trunk Rotation: Compensation during movement / unable to rotate shoulder to mid-line		

CERVICAL SPINE MOVEMENTS

CHECKPOINT	COMPENSATION	RIGHT	LEFT
**VIEW: FRONT**			
Spine	Lateral Flexion: Compensation during movement / unable to side-bend half the distance to shoulder		
	Rotation: Compensation during movement / unable to rotate chin to shoulder		

NOTES	ASSESSMENT	PLAN

Practitioner Printed Name: ______________________ Practitioner Signature: ______________________
